# Decades Delayed in Diagnosis: Hidradenitis Suppurativa and a Review of Barriers to Care

**DOI:** 10.7759/cureus.56231

**Published:** 2024-03-15

**Authors:** Hannah Riva, Aleksi Hendricks, Teresa Yoon, Carlos Del Coro Amengual, Craig Maddox

**Affiliations:** 1 Medical Education, Texas Tech University Health Sciences Center Paul L. Foster School of Medicine, El Paso, USA; 2 Dermatology, University of Arizona, Tucson, USA; 3 Surgery, Las Palmas Medical Center, El Paso, USA; 4 Dermatology, Mountain View Dermatology, El Paso, USA

**Keywords:** general surgery, dermatology, delayed diagnosis, barriers to care, hidradenitis suppurativa

## Abstract

We present a case of a 40-year-old female seen on the inpatient general surgery service in consultation for a suspected abdominal wall abscess or seroma. The history and examination were consistent with a diagnosis of hidradenitis suppurativa. The patient had a 25-year history of similar lesions present since her teenage years, not properly investigated and diagnosed, despite presenting with symptoms in multiple clinic and hospital settings since disease onset. As an accurate diagnosis of HS is often missed or delayed for years, it is important to increase awareness and clinical recognition of this condition among providers to improve outcomes for patients with the potentially debilitating disease of HS.

## Introduction

Hidradenitis suppurativa (HS) is an inflammatory, often debilitating, dermatosis with a predilection for the intertriginous areas of the axillary, perianal, inframammary, inguinal, and perineal regions. HS involves the folliculopilosebaceous units and is thought to involve follicular occlusion that leads to recurrent inflamed nodules or abscesses; these can progress to form draining tunnels and scarring, causing pain, disfigurement, debility, and significant psychological distress. Disease onset is usually in the second or third decade, and estimates of HS prevalence range from less than one to four percent [1,2]. The incidence of HS in women was found in one population-based study in the United States to be twice that in men, and the incidence in African Americans in this study was over 2.5 times that of non-Hispanic whites (NHWs) [3].

The Hurley staging system is used to grade the severity and activity of HS disease lesions. In Stage I, inflammatory nodules or abscesses form without skin tunnels or scarring. In Stage II, draining skin tunnels and scarring form. In Stage III, there is diffuse involvement of affected regions, or multiple connected skin tunnels and abscesses completely spanning the involved area. Hurley staging has been found to be reliable for rapidly assessing active inflammatory lesions and the severity of HS [4]; however, its ability to quantify the extent or physical impact of scarring is limited.

Factors associated with the development of HS include obesity, type 2 diabetes, and cigarette smoking; hormones, insulin resistance, and nicotine may also affect the follicular epithelium [2]. Genetic susceptibility also appears to play a role in HS, as approximately 40% of HS patients have a first-degree relative with HS [5]. Diagnoses that may be associated with HS include metabolic syndrome, inflammatory bowel disease (particularly Crohn’s disease), acne vulgaris, and other follicular occlusive diseases such as pilonidal sinus, acne conglobata, and dissecting cellulitis of the scalp. HS can significantly adversely affect patients’ quality of life, including psychosocial distress in body image and sexuality, as well as an increased risk of depression, anxiety, and suicide [6,7].

## Case presentation

A 40-year-old Hispanic female with rheumatoid arthritis and type 2 diabetes mellitus was consulted by the inpatient general surgery service for a suspected abdominal wall abscess, supported by a left lower quadrant abdominal wall ultrasound report indicating a likely seroma measuring approximately 3 × 3 cm. She reported experiencing three months of drainage from a “cyst” containing malodorous yellow or brown serosanguineous fluid at her left infra-abdominal fold. Additionally, she noted a history of similar lesions in the bilateral inguinal folds, folds between the groin and inner thighs, axillae, and inframammary folds, with episodes of draining lesions dating back to age 15. She was diagnosed with type 2 diabetes at the age of 15. She had been evaluated by several providers due to complaints of similar draining nodules and reported that she was never given a formal diagnosis. Additionally, she mentioned a two-year history of occasional cigarette smoking from ages 27 to 29 but stated that she ceased smoking entirely after that period.

**Figure 1:**
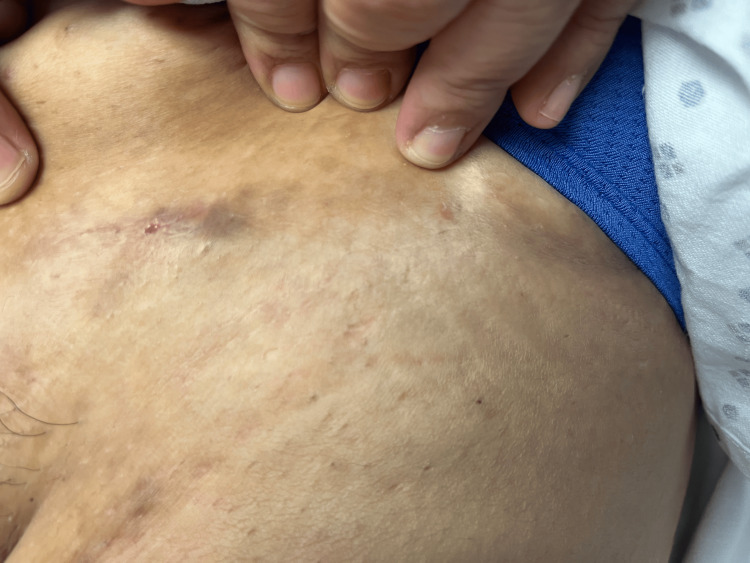
A 1-centimeter, tender, superficial subcutaneous tracking nodule draining serous fluid in the left infra-abdominal fold

**Figure 2:**
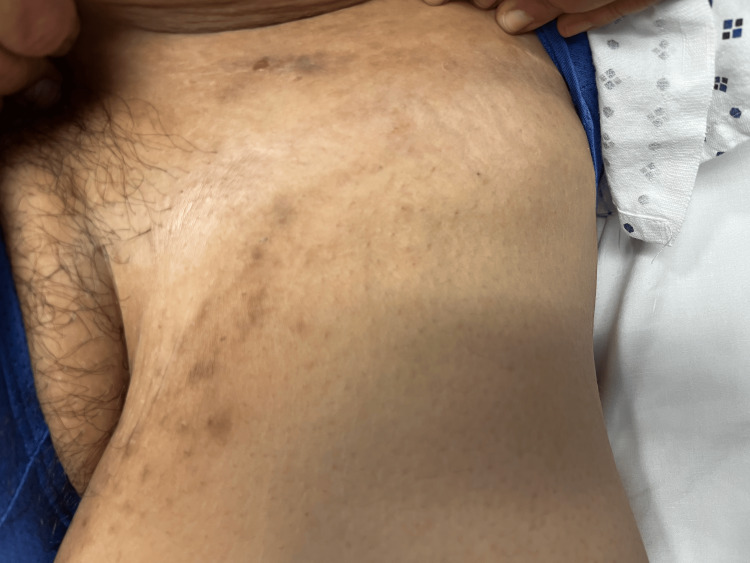
Hyperpigmented stranding fibrotic scars in the superior inner thigh region near the groin

**Figure 3:**
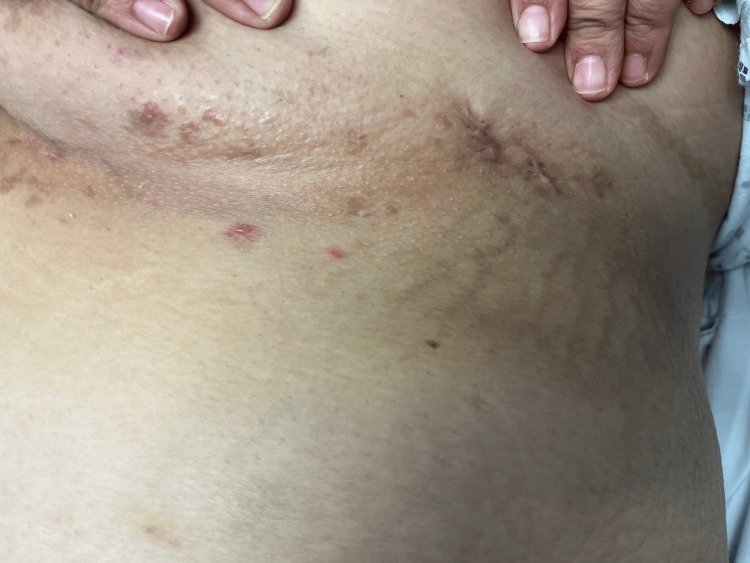
Additional hyperpigmented stranding fibrotic scars in the inframammary folds

On examination, the body mass index was 47.9. A 1-centimeter, tender, superficial subcutaneous tracking nodule was observed in the left infra-abdominal fold (Figure 1). Scant serous fluid was expressed with pressure. Further examination revealed hyperpigmented stranding fibrotic scars in the bilateral inguinal folds and superior inner thigh regions near the groin (Figure 2), as well as in the inframammary folds (Figure 3).

The history and examination of the draining nodule in the infra-abdominal fold, as well as involved areas of the inguinal and inframammary folds and axillae, were consistent with a clinical diagnosis of Hurley Stage II HS. Despite experiencing similar symptoms for over 25 years, this was the patient’s first diagnosis of HS. The patient had never seen a dermatologist and expressed the belief that her insurance coverage was a barrier to dermatologic evaluation. Furthermore, our region is profoundly underserved with regard to medical specialties, including dermatology. To help her obtain access to dermatologic care, she was referred to a board-certified staff dermatologist at a local volunteer clinic, where an appointment was scheduled for her for dermatologic consultation and necessary follow-up care.

## Discussion

Delays in diagnosis of HS are associated with more severe disease and poorer outcomes. Patients with delays in diagnosis are more likely to have a severe stage of disease at diagnosis, a disease stage requiring surgical intervention, and an increased work disability and impairment of professional life [8]. Our patient already had areas of Hurley Stage II HS, notably scarring in multiple areas, at the time of diagnosis.

Treatment of Hurley Stage I HS or mild disease includes topical or oral antibiotics, intralesional corticosteroids, and possibly antiandrogenic hormonal agents. For Stage II or III HS or moderate-to-severe disease, tumor necrosis factor alpha inhibitors or an interleukin 17A inhibitor may be indicated, and surgery may be required at this stage.

The average duration from symptom onset to diagnosis of HS is estimated at around four to 10 years [8-10]. Factors associated with delays in diagnosis of HS include younger age at onset, non-smoking status prior to HS onset, and late or no dermatologic consultation [8]; all three of these factors were present in our patient. Delay in or lack of dermatology consultation is also more common in geographic areas with limited dermatologists as well as in populations lacking financial resources and insurance commonly accepted by private practice clinics. HS is associated with increased disease severity in patients with skin color [11]. Hispanic patients have also been shown to have longer delays in the diagnosis of HS compared with NHW patients [10]. Further, Hispanic patients have, on average, significantly less access to dermatologic care than their NHW counterparts [12].

There is a need for further analysis of the reasons for delays in the diagnosis of HS as well as education for healthcare providers to clinically recognize the condition and refer appropriately. A recent educational campaign on HS for clinicians has been undertaken in Spain; this study identified a lack of HS training among general practitioners and implemented an educational training tool about HS via the WhatsApp platform [13]. The study found that a third of general practitioners did not believe that patients could benefit from a referral to a dermatologist. HS encounters occur most commonly with family or internal medicine providers (rather than with dermatologists), and encounters with these providers involve frequent opiate prescriptions, low use of dermatology referrals, and low use of nonantibiotic systemic therapy [14].

There remains a significant opportunity for bridging the gap in general knowledge among healthcare practitioners about HS in order to be able to recognize, diagnose, and refer appropriately to facilitate prompt and adequate treatment for patients with this challenging condition.

## Conclusions

This case highlights the critical importance of clinical recognition and diagnosis of HS and coordination of care between general practitioners and dermatologists in order to expedite the treatment of HS and the psychosocial challenges it can present for patients. Factors associated with delays in the diagnosis of HS include younger age at onset, absent or late dermatologic consultation, and non-smoking status prior to HS onset. In later diagnoses, there is often a more severe disease stage at diagnosis, a higher likelihood of receiving surgery, and an increased work disability and impairment of professional life. As the accurate diagnosis of HS is often missed or delayed for years, it is important to increase awareness and clinical recognition of this condition among providers to improve outcomes for patients with the potentially debilitating disease of HS.
